# A systematic review of instrumented assessments for upper limb function in cerebral palsy: current limitations and future directions

**DOI:** 10.1186/s12984-024-01353-6

**Published:** 2024-04-16

**Authors:** Julie Rozaire, Clémence Paquin, Lauren Henry, Hovannes Agopyan, Rachel Bard-Pondarré, Alexandre Naaim, Sonia Duprey, Emmanuelle Chaleat-Valayer

**Affiliations:** 1 Service de Médecine Physique et de Réadaptation, Centre Médico-Chirurgical de Réadaptation des Massues Croix-Rouge française, Hôpital de Jour, Lyon, France; 2 LBMC UMR_T9406, Univ Lyon, Univ Gustave Eiffel, Université Claude Bernard Lyon 1, Lyon, France; 3 Texisense, Torcy, France

**Keywords:** Cerebral palsy, Upper limb, Motion analysis, Instrumented assessments, Clinical applicability

## Abstract

**Introduction:**

Recently, interest in quantifying upper limb function in cerebral palsy has grown. However, the lack of reference tasks and protocols, have hindered the development of quantified movement analysis in clinical practice. This study aimed to evaluate existing instrumented assessments of upper limb function in cerebral palsy, with a focus on their clinical applicability, to identify reasons for the lack of adoption and provide recommendations for improving clinical relevance and utility.

**Methods:**

A systematic review was conducted by a multidisciplinary team of researchers and clinicians (Prospero CRD42023402382). PubMed and Web of Science databases were searched using relevant keywords and inclusion/exclusion criteria.

**Results:**

A total of 657 articles were initially identified, and after the selection process, 76 records were included for analysis comprising a total of 1293 patients with cerebral palsy. The quality assessment of the reviewed studies revealed a moderate overall quality, with deficiencies in sample size justification and participant information. Optoelectronic motion capture systems were predominantly used in the studies (N = 57/76). The population mainly consisted of individuals with spastic cerebral palsy (834/1293) with unilateral impairment (N = 1092/1293). Patients with severe functional impairment (MACS IV and V) were underrepresented with 3.4% of the 754 patients for whom the information was provided. Thirty-nine tasks were used across the articles. Most articles focused on unimanual activities (N = 66/76) and reach or reach and grasp (N = 51/76). Bimanual cooperative tasks only represented 3 tasks present in 4 articles. A total of 140 different parameters were identified across articles. Task duration was the most frequently used parameter and 23% of the parameters were used in only one article.

**Conclusion:**

Further research is necessary before incorporating quantified motion analysis into clinical practice. Existing protocols focus on extensively studied populations and rely on costly equipment, limiting their practicality. Standardized unimanual tasks provide limited insights into everyday arm use. Balancing methodological requirements and performance evaluation flexibility is a challenge. Exploring the correlation between outcome parameters and therapeutic guidance could facilitate the integration of quantified movement assessment into treatment pathways.

**Supplementary Information:**

The online version contains supplementary material available at 10.1186/s12984-024-01353-6.

## Introduction

Cerebral palsy (CP) is the most common motor disorder in children in most countries, with an estimated prevalence of 17 million affected people worldwide [1]. CP is a group of lifelong neurological disorders caused by damage to the developing brain during pregnancy, childbirth, or shortly after birth. It affects motricity, cognition and sensorial integration [2]. CP is a non-progressive disorder but functional impairments may change over time, underscoring the importance of regular assessments to monitor and adjust treatment plans accordingly.

For lower limbs, gait analysis has become an important tool to quantify locomotor disorders and plan treatment [3, 4]. Walking is a fundamental task that represents the daily use of the lower limbs, and has therefore been extensively studied and described [5–7]. However, the analysis of upper limb movements is more complex due to the greater number of degrees involved and the variety of tasks that can be performed. Indeed, the upper limbs play a crucial role in moving, stabilising, and manipulating objects in the environment. Bimanual actions are essential for activities of daily living such as feeding, dressing, toileting and personal hygiene, as well as specific learning tasks that require even more developed fine motor skills, dexterity and precision. In order to develop effective interventions that can improve patient's ability to participate in meaningful occupations and promote well-being, it is essential to conduct a comprehensive assessment of the different domains of the International Classification of Functioning, Disability and Health (ICF) [8]. Thus, occupational therapists use a variety of standardized and patient-specific assessment tools to evaluate those different domains of the ICF, and tailor interventions based on the individual's unique needs and goals. These assessment tools often involve the use of questionnaires [9, 10] or direct/video-based observation to score the range and quality of movement during task performance [11–13]. However, these assessments can be biased due to the subjective nature of the evaluator's visual assessment and the limited amount and type of information available to the eye.

Consequently, there has been a growing interest in using instrumented assessments to provide a more objective and quantitative evaluation of upper limb function in individuals with CP. Although the first attempts to record and describe the upper limb during a reaching task date back a century [14], the use of upper limb movement analysis in clinical practice remains limited. According to a recent international survey, the main barriers to the use of quantified upper limb motion analysis in clinics are the availability of standard reference tasks, protocols, software, funding, and clinical need [15]. The authors emphasised the need for a clear link between impairments, required biomechanical data and clinical outcomes/interventions in order to achieve wider use of motion analysis. Although some correlations have been made between kinematics and impairments or clinical outcomes [16], as well as between clinical outcomes and impairments [17], clear guidelines linking impairment identification to interventions are still to be established.

Building on the conclusions of Philp et al. [15] stating a major lack of adoption of upper limb motion analyses in the clinical context, this review aims to analyse the literature to understand the reasons for this lack of adoption and provide recommendations to improve the clinical relevance and utility of instrumented assessments for upper limb function in CP.

## Methods

A protocol describing the current review has been recorded and published on the PROSPERO register database, registration number: CRD42023402382. This review is reported according to the PRISMA guidelines.

### Search and selection

A comprehensive search of the electronic databases PubMed and Web of Science was performed in September 2022. The search blocks were linked with *AND* logical bond and included: the diagnosis with “*Cerebral Palsy OR Dyskinesia OR Dystonia OR Spasticity”*, the body region with “*Upper OR Arm OR Wrist OR Elbow OR Shoulder OR Finger OR Forearm OR Hand”* and the measurements with keywords such as “*Biomechanics OR Kinematic OR Motion Analysis OR Spatiotemporal OR 3D”*.

Identified articles were transferred to Rayyan (Qatar Computing Research Institute, Qatar). Duplicates identified by Rayyan were individually checked to be deleted. Two reviewers with different backgrounds (JR an engineer, CP a physiotherapist) independently screened titles and abstracts against the inclusion criteria. The inclusion criteria are presented in Table 1 using the PICOS framework (Participants, Intervention, Comparison, Outcome, and Study design) [18]. In case of any disagreement on inclusion or exclusion, the articles were read in full and a discussion between reviewers led to a decision on their classification.

**Table 1:** PICOS framework for the definition of inclusion and exclusion criteria

	Description	Inclusion/exclusion criteria
Participants	Patients diagnosed with cerebral palsy over the age of 4	• At least half of the study group were composed of patients with cerebral palsy older than 4 years of age • Articles on athletes were excluded
Intervention	Instrumented measurements to assess upper limbs function by measuring kinematic parameters	• Articles focusing only on passive movement were excluded • Articles using only EMG, force measurements or MRI techniques were excluded • Articles using video recording without computerizing analysing techniques were excluded
Comparison	No comparison group is required	• Comparison methods were not reported but are not a requirement for inclusion
Outcome	Outcomes measured in one of the following ICF^8^ levels: muscle or movement functions, carrying, moving and handling objects, (fine) hand and arm use	• Articles focusing only on the head or the trunk movements were excluded • Articles on standing or sitting position and walking tasks were excluded • Articles studying robots were excluded
Study design	Full-text articles	• Articles written in a language other than French or English were excluded • Opinion letters or conference abstracts were excluded

### Quality assessment

A customized checklist of 20 questions was developed based on existing systematic reviews in the field of biomechanics [19–21] to assess the methodological quality of the studies included in the present review. Each question was initially rated zero (missing information) or one (information provided) by the primary reviewer (JR), and then the articles were rated by another reviewer (LH an engineer) for a second rating to ensure consistency and quality. In case of conflicts, a third reviewer (CP) served as an arbitrator. The quality criteria were grouped into five categories: “Methodology”, “Study Design”, “Population”, “Reliability”, and “Discriminatory Power and Ecological Validity” (Table 2). In order to balance these different categories according to their importance, a weight has been assigned to each category. The total score is the sum of the averages per category times their weight given out of 20. Studies that received a final score lower than 10 were excluded from the review.

**Table 2:** Quality assessment criteria gathered in categories

Category (Weight)	N°	Question
Methodology (3)	C1	Are the objectives of the research clearly defined?
	C2	Are the kinematic outcomes linked with the research objectives?
	C3	Is the sample size justified?
	C4	Is the statistical analysis detailed?
	C5	Are the results linked to clinical measurements (presence of at least one clinical assessment in the outcomes)?
	C6	Are the limitation of the study described?
	C7	Are the results linked to other outcomes in the literature?
Study design (3)	C8	Is the installation of the participant well described (sitting condition, position at rest)?
	C9	Are the tasks reliably described so that they can be reproduced?
	C10	Are the assessment tools clearly described? (for motion tracking system, brand, acquisition frequency and markers location will be expected)
	C11	Are the outcome parameters clearly defined, enough to be recalculated?
Population (3)	C12	Is the most impaired side of the participants given?
	C13	Is the dominant type of cerebral palsy described (e.g.: spastic, dyskinetic, ataxic)?
	C14	Is the functionality of the upper limbs described for the group of participants or for each participant?
Reliability (2)	C15	Is the test–retest/intra session repeatability studied?
	C16	Is the inter-session repeatability studied?
	C17	Is the inter-rater repeatability studied?
	C18	Is the sensitivity to change studied?
Discriminatory power and ecological validity (1)	C19	Do the parameters utilized enable discrimination between distinct functional levels of impairment?
	C20	Does the task resemble to real-life situations (e.g.: Reach and grasp to drink from a cup, playful environment, bilateral box picking up)?

### Data extraction

Items to be extracted were first selected by the first reviewer (JR), then those items were reviewed by a second reviewer (CP) to build a custom-made Excel (Microsoft Office, Microsoft, Redmond, WA, USA) data extraction form. JR carried out the extraction for all articles, then CP and LH shared articles for a second extraction to ensure the quality of the extraction by the first reviewer. In case of conflicts, the third reviewer served as an arbitrator.

The final data-table was built using the following data items: articles information (first author, year of publication, title, country), population characteristics (number of subjects with CP, age, impairment type, Manual Ability Classification System-or MACS-levels [22] and Gross Motor Function Scale-or GMFCS-levels [23] and number of typically developing children included if applicable), measurement system, the tasks description (unilateral or bilateral, type of task and general description) and the parameters used in the study. This data-table is available as a supplementary material (Additional file 2). As the description of the tasks was incomplete in several articles, some information about the task description was extracted from the photos illustrating the experiments. When available, moderate to excellent significant correlations between kinematic parameters and clinical outcomes for given tasks were also reported in a second data-table available as a supplementary material (Additional file 3). The significance threshold is 5% and a correlation is considered moderate if the correlation coefficient is greater than 0.5 [24].

**Fig. 1:**
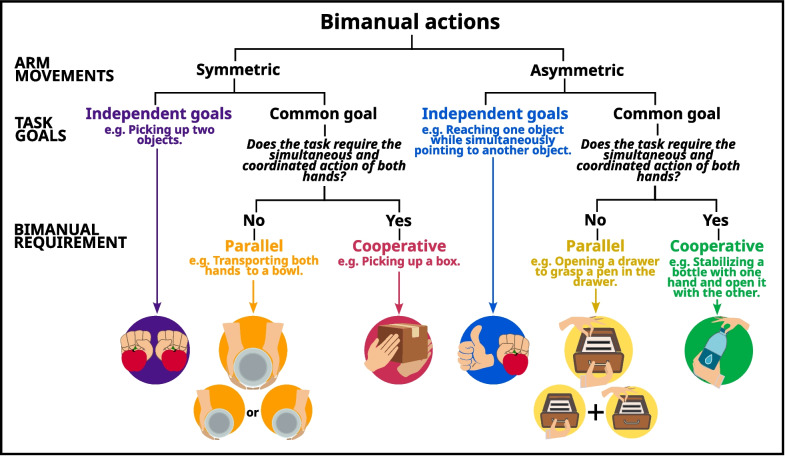
Classification of bimanual actions adapted from Kantak et al*.* [25]

To discriminate between the different kind of bimanual tasks, the classification of bimanual actions was based on the symmetry of arm movements and conceptualization of task goals defined by Kantak et al*.* [25] was used (Fig. 1). This classification is a valuable tool for understanding the complexity of bimanual tasks, dividing them into symmetrical and asymmetrical tasks, and distinguishing between tasks with independent versus common goals. Common goal tasks can be further classified into parallel tasks, which involve separate but coordinated actions towards a common goal (such as opening a drawer and taking something out), and cooperative tasks, which require coordinated action of both limbs to achieve the goal (such as cutting a steak with a fork and knife). For children with neurological impairments, asymmetrical tasks can be particularly challenging due to the need to overcome mirror movements, while cooperative tasks are especially important for fostering autonomy as they require coordinated action of both limbs.

Descriptive analysis of the extracted data was carried out according to the technology used, the type of population studied, the type of tasks analysed, the categories of parameters measured and their correlation to clinical outcome.

## Results

### Study selection

**Fig. 2:**
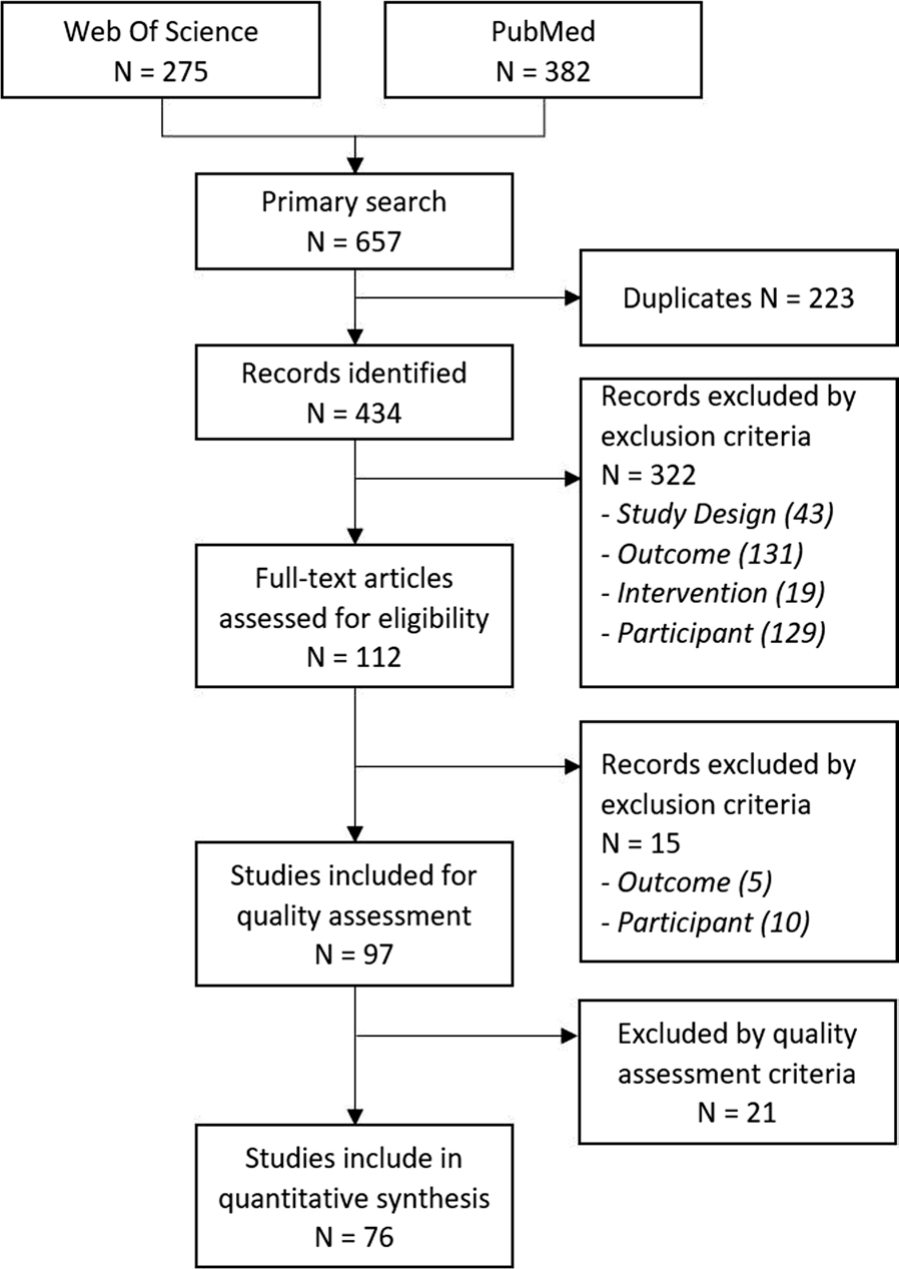
PRISMA flow diagram for identification and selection process

The selection process used to identify and select relevant papers is shown in the PRISMA flowchart in Fig. 1. A total of 657 articles were identified in PubMed and Web of Science; after removing duplicates, 434 articles remained to be screened and finally 97 records were included for analysis. Fifteen articles were excluded for the following main reasons: 1) half of the participants were too young or diagnosed with another pathology or 2) the assessment was not instrumented (e.g. uncomputerized video analysis) (Fig. 2).

### Quality assessment

Overall, the quality of the reviewed studies was moderate, with a mean score of 10.9 ± 2.1 out of 20 (Additional file 1). Only a small percentage of articles justified their sample size (5%) and a limited number of articles provided detailed information about participant installation (59%). In addition, half of the articles did not entirely describe the population, with only 54% of the studies reporting the type of CP and 63% mentioning the level of manual ability of the participants. Additionally, only 39% of the articles compared their kinematic results with clinical assessment findings, and only 20% used kinematic outcomes to distinguish between different levels of functional impairment. Twenty-one studies included for the quality assessment scored less than 10 and were excluded for the data extraction process. Finally, 76 studies were included in the quantitative synthesis with an mean quality score of 11.8 ± 1.5 [16, 26–98].

### Data extraction

**Fig. 3:**
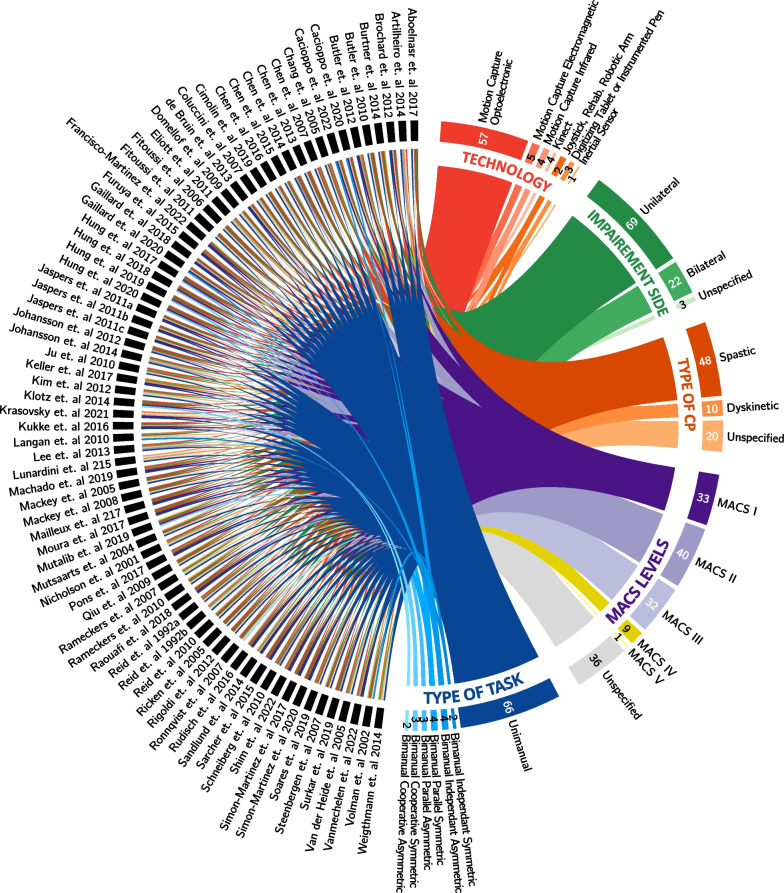
Links between studies, measurement tools, tasks, impairment side, CP sub-types, and MACS levels

To provide a comprehensive overview of the extracted data, the links between the included studies, their measurement tools, the type of tasks used, the side of impairment, the dominant type of CP, and the MACS scores of their participants was displayed on using a Circos plot [99] (Fig. 3).

### Technology

Most of the reviewed articles used optoelectronic motion capture systems (N = 57/76), which correlates with previous findings [15, 21, 100]. Two articles used the Kinect marker-less motion capture system, 1 article used inertial sensors, 3 used digitizing tablet/instrumented pens, and 4 articles used a joystick/robotic arm (Fig. 3).

### Population

**Fig. 4:**
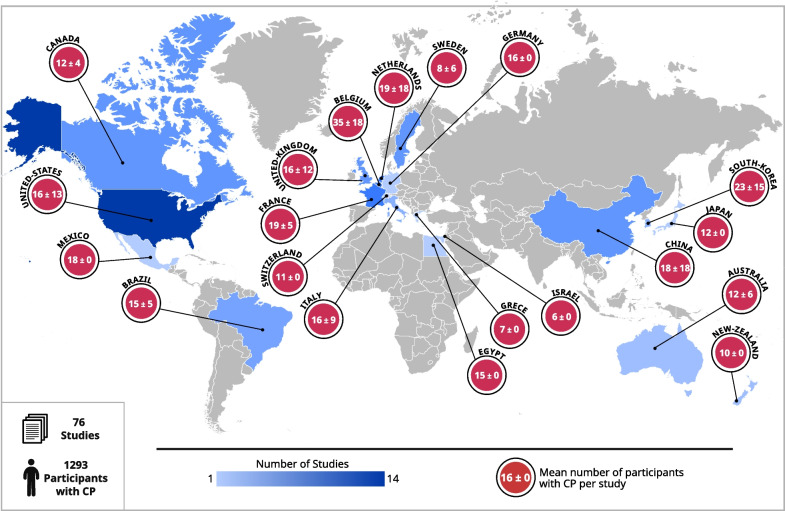
World map reporting the number of studies and participants with cerebral palsy per country

A total of 1293 patients with CP were included across the 76 articles from 20 different countries (Fig. 4). The population studied was mainly recruited in developed countries, especially in Europe. Most studies had small sample sizes, typically less than 20 participants.

#### Clinical types of CP

**Fig. 5:**
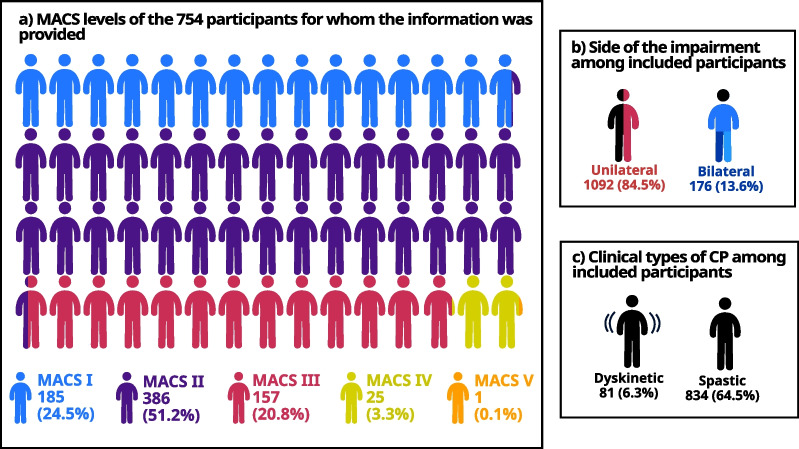
Participant distribution into subtypes of CP

Out of the 76 studies examined, 10 studies included individuals with dyskinetic CP (Fig. 3), representing 6% of the total population studied (N = 81) (Fig. 5). On the other hand, 48 studies included individuals with spastic CP, making up 65% of the total population (N = 834).

### Severity of the functional impairment

Patients with the lowest capacities, MACS IV and V children, are underrepresented in the included studies, respectively 25 participants over 9 studies and 1 participant in 1 study (Fig. 5).

#### Bilateral impairment

The reviewed studies showed that the majority of patients had unilateral impairments, with only 13.6% having bilateral impairments (N = 176) (Fig. 4). This is underlined by 69 of the 76 articles (67%) focusing on populations with unilateral impairment against only 22 for bilateral impairment (Fig. 3).

The population characteristics and measured parameters are presented in full in the (Additional file 2) to ensure comprehensive and transparent reporting of information.

### Tasks

#### Tasks laterality

Sixty-six of the 76 articles in this review examined unimanual tasks, while 11 articles used bimanual tasks to assess upper limb function in patients with CP. However, there are different forms of bimanuality that can be categorized according to Kantak's classification [25] (Fig. 1). Of these 11 articles, only 4 studies focused on cooperative tasks, with three different tasks: one being symmetrical, involving picking up a box with both hands, and the others asymmetrical, involving decanting cups or unlocking a door.

#### Tasks type

The different tasks of the articles were classified according to the objective of the action, the type of movement involved, and the location of the objectives. A total of 39 distinct tasks were found and are detailed in the Additional file 2.

To address the challenges of assessing the upper limbs, some researchers have chosen to study simple movements involving only one degree of freedom of a joint such as elbow flexion/extension, pronation/supination and abduction/adduction (N = 7/76). But, the majority of articles in the literature have investigated more functional tasks such as Reaching, and Reaching and Grasping (N = 51/76). Some authors examined Gross Motor Function tasks that simulate hygiene and feeding tasks such as “hand to head”, “hand to buttock”, “hand to contralateral shoulder” or “hand to mouth” similar to some Modified Melbourne Assessment (MA2) items (N = 17/76).

Finally, some articles studied activities of daily living (ADL) such as drawing, unlocking a locker, decanting cups, throwing a ball, picking up a box with both hands, and drinking or eating with a fork (N = 21/76).

### Parameters

#### Parameters categories

**Fig. 6:**
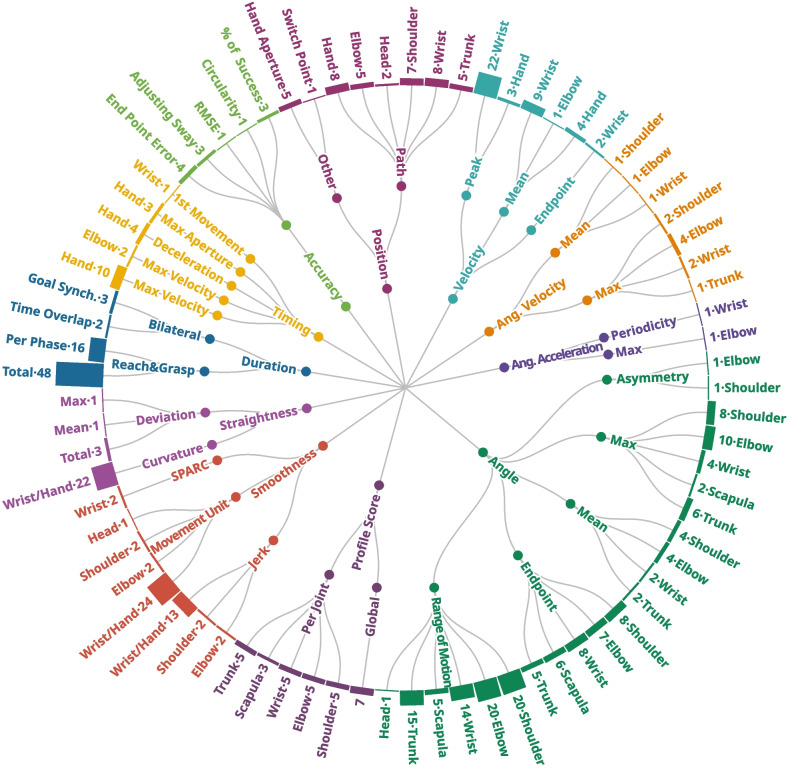
Overview of the parameters used in the articles

The analyzed articles identified a total of 140 different parameters, which can be categorized into several categories of parameters that offer valuable insights into various aspects of movement (Fig. 6).

The duration family concentrates on time-related factors, offering insights into the overall duration of a task or its specific components. In contrast, timing parameters provide information about the temporal aspects of movement events, such as when the peak velocity occurs within the task cycle. Smoothness parameters evaluate the fluency of movements based on acceleration metrics. The straightness family encompasses parameters that measure deviations from a straight line or the presence of curvatures in movement patterns. The position family includes parameters that describe joint displacement or provide distance-related information. Angle parameters focus on joint angles during movement. Velocity parameters specifically measure the linear velocities of particular joints, while angular velocity focus on the rotational speed, and acceleration captures both angular acceleration and a coefficient of periodicity of the acceleration profile. The accuracy family comprises parameters that assess the precision and accuracy of reaching or targeting tasks. The profile score family comprises parameters designed to quantify abnormal movement patterns compared to typically developing peers on the model of the Gait Profile Score used for gait [101], either overall on joint angles or for a specific joint. Figure 6 provides an overview of the parameters included in these categories. For a comprehensive list of the parameters and their occurrences in articles, please refer to the Additional file 2.

Notably, 23% of these parameters were found to be used only once, bringing the number of parameters that did not appear in any of the other articles to 32. The median number of articles using each parameter is 3 and the most frequently used parameter is the total duration of the task, which appeared in 48 articles.

### Correlation with clinical outcome

Out of the 36 articles that linked parameters to clinical outcomes (see criterion 5 of the Quality Assessment, Additional file 1), 14 found a moderate to excellent significant correlation [16, 31, 33, 39, 46, 53, 54, 59, 61, 64, 75, 78, 85, 89]. Two main articles have extensively studied the correlations between clinical outcomes and biomarkers, where a biomarker is the association between a task and a parameter. Among the 297 moderate to excellent significant correlations reported, 63% are from Mailleux et al*.* 2017 and 20% are from Jaspers et al*.* 2011b during unilateral reach and grasp and functional tasks [16, 46]. The detailed list of correlations can be found in Additional file 3.

The studies typically had small sample sizes, with a mean of 22.6 ± 12.2 patients included, and only 3 studies included more than 30 patients [16, 85, 89].

Only one excellent correlation was found between MACS levels and Total Movement Duration while eating with a spoon. The correlation coefficient was 0.912 and the p-value was less than 0.001 [61].

**Fig. 7:**
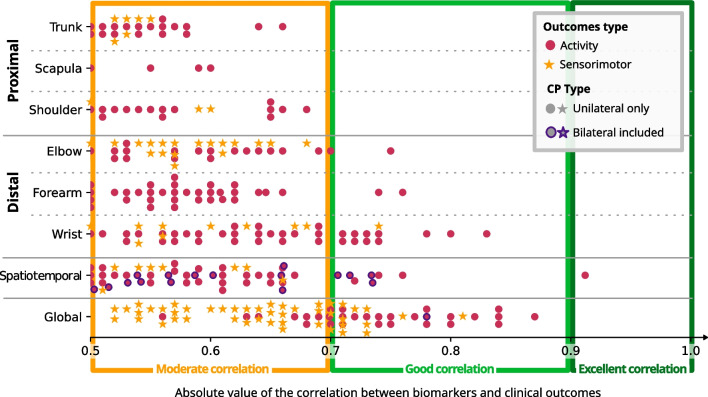
Absolute correlations between biomarkers and clinical outcomes sort by anatomical levels measured

Spatiotemporal parameters were measured at the wrist, hand, or finger level. Therefore, no good correlations were found at proximal anatomical levels such as the shoulder, scapula, or trunk, as shown in Fig. 7.

Most studies have only focused only on patients with unilateral cerebral palsy. The three studies that included bilateral participants showed moderate to good correlation with clinical outcomes, but did not examine specific joint kinematics [31, 59, 89].

All studies correlated the parameters with activity outcomes, and two studies also correlated them with sensorimotor outcomes [16, 46].

## Discussion

The current review provides an overview of the available instrumented assessments of upper limbs function in CP. As previously noted by Philp et al*.* [15], there is a wide range of study designs available fulfilling various goals. Many articles did not provide enough detail about the characteristics of the participants or their installation, making it difficult to replicate or compare their results.

### Technology

As mentioned above, the most commonly used measurement tool is a marker-based motion capture system. This technology is a reference measure for capturing a large number of parameters related to a person's movement and posture. However, motion capture systems, especially optoelectronic ones, can be quite expensive. In addition, the need for skilled operator to process the data and accurately place the markers on the patient's body can limit their use in clinical settings. The use of markers can also be invasive, as it may require the patient to undergo the experiments shirtless in a non-ecological environment. Two studies used Kinect markerless systems (Microsoft, Redmond, Washington, U.S.), which are a low-cost option for capturing patient movement data without the need to place markers, compared to marker-based technology, although with lower accuracy [102]. The main promise of such technologies is the possibility to perform motion capture with the possibility for the participant to remain clothed, thus taking less time and being minimally disrupting [103]. The Kinect technology utilises depth sensors to track body displacement. However, this technology has limitations on capture rate and volume, and data collection may require controlled lighting conditions [104]. Other markerless technology based on pose estimation from RGB video have been developed since [105, 106]. However, their reliance on an artificial intelligence algorithm trained on typically developing patients raises questions about the applicability of such technology on pathological participants.

The investment in motion capture systems can be rewarding, as they have demonstrated their usefulness in informing cost-effective surgical decisions for lower limbs. However, the complex nature of upper limb movements, characterized by greater degrees of freedom and a wider variety of tasks, poses a significant challenge in applying motion capture outcomes to achieve similar advancements in multi-site surgery for the upper limbs. This highlights the pressing need for further progress in this area.

The remaining articles chose to instrument the object used during the task rather than the participant, using inertial sensors, joysticks, robotic arms or a digitising tablet. These approaches are less invasive as they do not require the participant’s body to be instrumented. However, these approaches are task-specific and cannot capture the full range of the participant’s movement and posture that other technologies can.

This study prioritizes instrumented assessments that provide objective measures of movement characteristics through spatiotemporal or kinematic parameters. It is important to acknowledge that this focus represents a limitation. Other types of instrumented assessments, targeting different constructs, could provide valuable insights into a patient's progress and would require a separate review [107, 108]. For instance, body-worn sensors like accelerometers, gyroscopes or inertial measurement units (IMUs)—which combine gyroscopes, accelerometers and sometimes magnetometer -, offer a compact and low-cost option for measuring activity outside the clinical setting. In patients with CP, accelerometers have shown success in measuring changes in upper limb use among toddlers after constraint-induced therapy in daily life [109]. However, their reliability in infants under one year remains uncertain [110]. Similarly, IMUs demonstrate promising reliability in recognizing upper limb movements and manual activity during activities of daily living in stroke survivors [111–114]. As sensor technology advances in miniaturization, computation, and communication, opportunities for measuring performance in daily life settings will continue to expand [107].

When considering the clinical applicability of a tool, it is essential to evaluate its financial, human, and logistical costs. Motion capture systems, while being state-of-the-art tools, come with their own set of challenges as they are expensive and require expertise and time-consuming setup for marker-based versions. However, from a research perspective, motion capture systems prove to be versatile tools capable of measuring a wide range of parameters in both proximal and distal joints, enabling laboratories to gain insights into the movement patterns of patients with CP. Notably, these systems have contributed to reducing the number of surgeries in lower limbs, though further studies are needed to achieve similar outcomes for upper limbs. For widespread clinical use, the next steps involve identifying the relevant parameters based on the clinical question and finding accurate, minimally invasive, and cost-effective methods to measure these parameters.

### Population

CP is a complex condition that encompasses a wide range of impairments and unique patient profiles, leading some experts to refer to it as the CP spectrum [115]. Each patient's CP subtype might require a different approach to analysis and treatment. Therefore, accurate analysis and effective therapeutic interventions require context-specific interventions that consider the CP subtype. For example, trunk range of motion may be considered a compensatory strategy in a patient with spasticity, whereas in a dyskinetic patient it may be due to a dystonic behaviour. However, during the quality assessment of the reviewed articles, only 55% of the studies correctly identified the type of CP of the participants and only 64% of the articles described the functional level of the participants. In order to establish links with underlying causes and to develop effective therapeutic interventions, future studies would strongly benefit from a better detailing of the pathological characteristics of their participants, which would improve the clarity and reproducibility of their findings.

### Clinical types of CP

Of the participants included in this review 6% had dyskinetic CP, this accurately reflects the proportion of dyskinetic patients within the wider CP population [116]. For a more comprehensive evaluation of the instrumented assessments available for individuals with dyskinetic CP, Haberfehlner et al*.* [100] conducted a systematic review of outcome parameters to measure the control of voluntary and involuntary movements.

Recently, Ralph et al*.* [117] proposed a framework for assessing patients with dyskinetic movement disorders, highlighting the current lack of tools to accurately measure choreoathetosis. The Dyskinesia Impairment Scale [13] is currently the only tool available for this purpose, but it requires considerable time and clinician expertise to administer it and score accurately. Therefore, there is an urgent need for the development of easy-to-use, less time-consuming tools which can differentiate between dystonia and choreoathetosis. Advanced quantitative motion analysis techniques hold promise in addressing this challenge, as they have already been successfully used to differentiate between different types of chorea [118].

### Severity of the functional impairment

As noted above, CP patients with lower functional levels, such as those classified as MACS IV or V, are often under-represented in instrumented studies. This may be because patients with the most severe functional impairment are more likely to have cognitive problems [119], which may lead to their exclusion from trials. Unfortunately, these patients are also excluded from the only existing clinical tools that assess bimanual performance; the Assisting Hand Assessment (AHA) [12] and the Both Hand Assessment (BoHA) [120].

These patients have limited ability to manipulate objects and perform activities of daily living, so assessments often focus on how caregivers can help them with basic daily activities such as dressing and washing. However, it is important to note that the remaining abilities of this population are critical to their independence and quality of life. These limited but existing capacities, such as the ability to rake objects to themselves, to use a smartphone or to operate an electric wheelchair, should not be overlooked and should be further investigated in future studies.

### Bilateral impairment

In the included studies, 84.5% of patients with CP had unilateral impairment, which does not reflect reality as approximately 60% of patients with CP have bilateral impairment [121]. However, this finding is consistent with previous research [21] and can again be explained by the large number of quadriplegic patients with severe cognitive impairment [122]. Yet, patients with bilateral CP are also insufficiently studied using existing clinical tools. This lack of research has led to a significant gap in our understanding of the effectiveness of interventions for this group [123, 124], which is likely due to the lack of appropriate outcome measures [125]. Therefore, further research is needed to develop and validate outcome measures that can accurately assess the impact of interventions on patients with bilateral impairments.

The limited attention given to upper limb function in patients with bilateral CP can also be attributed to the fact that treatment often primarily focuses on lower limb impairments. This impairment holds greater significance in treatment as it is frequently identified by parents as their primary concern [126, 127]. However, when it comes to children severely affected by CP (GMFCS IV or V), parents often express concerns regarding the mobility domain, which is predominantly influenced by manual ability.

In summary, the existing literature often lacks detailed descriptions of the type of CP being studied. Furthermore, when such descriptions are provided, they tend to focus on the least affected individuals, such as those with unilateral impairment, good functional capacity and no dyskinesia, although this population is already well documented in the clinical literature. Clinicians lack tools for the more complex forms of CP, where limited capacity, bimanual coordination or abnormal movements may be difficult to monitor without instrumentation.

### Tasks

The task to be assessed is a crucial element of the clinical evaluation, as it must be clinically relevant and representative of the upper limb's use in daily life, while also being repeatable to ensure consistent measurement.

### Tasks laterality

In everyday life, most of the activities require a bilateral use of the arms [128–130]. However, the majority of the tasks used to assess upper limb function in children with CP are unilateral. The study of bimanual coordination is challenging but it is necessary to gain insight on how to better capture the performance of children with CP in everyday life. Cacioppo et al*.* [21] recently published a review of the use of instrumented measures tools during bimanual tasks in children with CP. They provided a comprehensive overview of the parameters commonly used to describe bimanual movement and successfully analysed their metrological properties. However, 'bimanual' is a broad term and some bilateral use of the arms is more challenging for patients with neurological impairment.

Indeed, asymmetric tasks, as described by Kantak et al*.* [25], can be more difficult for patients with neurological impairment, and that cooperative tasks are the only ones in which the completion of the task depends on the coordinated use of both limbs. These tasks are also the least studied in the literature, although there are many examples of cooperative tasks in everyday life: opening the cap of a bottle, opening a tube of toothpaste or cutting meat with a knife and fork.

The AHA and the BoHA focus primarily on cooperative tasks. Their instrumentation would therefore allow the study of inter-limb coordination and other kinematic parameters during cooperative tasks in parallel with a clinical examination. Although, these clinical instruments have been developed with the main concern of being able to provoke the spontaneous behaviour of the patient, in terms of upper limb use. Thus, the important question arises as to whether it is possible to add instrumentation to the test administration; that is to say, whether it is possible to measure the real performance in an instrumented, i.e. non-ecological, way.

### Tasks categories

Some authors have used simple, isolated movements similar to study joints active and passive range of motion. However, the majority of studies in the literature have focused on assessing upper limb function using more complex, functional tasks such as reaching, grasping, gross motor tasks and activities of daily living. These tasks are more representative of real-life situations, although they are administered in a standardised manner to ensure reproducibility.

A major challenge in assessing upper limb function is that individuals, even within the typically developing population, may use different strategies to perform the same task. To make meaningful kinematic comparisons between individuals, researchers often constrain movements in favour of a particular way of reaching, grasping or manipulating objects. However, this approach may be at the expense of information about the patient's performance in everyday life.

Although assessing patients' abilities in a controlled environment is a crucial first step, it is only by assessing their performance that therapists can get a clear picture of the effectiveness of therapeutic interventions in everyday life. Indeed, there is almost always a gap between what the child can do (capacity) and what they actually do in everyday life (performance) [131, 132]. However, measuring performance in an uncontrolled environment is technically challenging. Furthermore, upper limb analysis is currently heavily influenced by the gait analysis model, with a desire for corridors of normality, joint kinematic curves or a cyclical approach. As a result, there is a need to consider new approaches that are tailored to the specific needs of upper limb analysis and can capture the complexity of real-life activities and the efficiency of individual strategies. This means including more cooperative bimanual tasks and protocols which allow patients to express more than one functional strategy. However, it's important to note that the choice of tasks to be assessed should always be closely linked to the clinical question and the patient's goals.

### Parameters

#### Parameters categories

The analysis revealed a wide range of parameters, many of which were used only once, making it difficult to compare results between research teams. In addition, the comparison and analysis of parameters obtained through different tasks, particularly angular values, is difficult and the wide variety of task types identified in this study makes any comparison even more difficult.

Measuring upper limb movement outcomes can provide clinicians with valuable and objective information about one’s motor impairment, assisting treatment planning. Each parameter family can be associated with specific clinical constructs and linked to different intervention strategies. For instance, smoothness parameters can measure abnormalities in tone control, suggesting treatments such as pharmacological agents or neurosurgical procedures to reduce tone [123]. Similarly, analysing movement straightness provides insights into the linearity and efficiency of movements, which can be addressed through training-based interventions to target motor impairment and compensatory movements. Additionally, depending on the context, angular results can inform the development of interventions such as splinting, surgical procedures, or pharmacological treatments to help enhance or decrease the range of motion in joint angle.

### Correlation with clinical outcome

Knowledge of the relationship between 3D motion analysis parameters and clinical scales improves our understanding of both the scales and the pathology, and relates clinical meaning to objective parameters. Although studies addressing this question are scarce, some authors have tackled this question, mostly in unilateral CP patients with grasping ability.

The primary limitation of most studies is the small sample size. Indeed, even in studies with larger populations, because CP is a highly heterogeneous condition, statistical analysis is limited by the number of patients per subtype. As calculated by Shim et al., if the statistical power is 80% and the expected correlation coefficient value is 0.5 in the MACS level III group, the required sample size is 29 in this category only, although most studies in this review recruited less than 20 participants. This would explain the small number of studies having moderate to excellent correlation [89].

As previously noted by Mailleux et al*.*, Gaillard et al*.*, and Jaspers et al*.* for the MA2, AHA, and House classification [133], it appears that usual clinical outcomes mostly capture distal motor function [16, 46, 53]. This is not entirely unexpected, given that the majority of AHA items (‘reach and grasp’ items, ‘fine motor adjustment’ items and ‘readjust grasp’ item) and half of the MA2 items (reach, grasp and release items, ‘prono/supination’ item, ‘manipulation’ item) are focused on distal behavior. Additionally, the House classification was developed to assess function in the affected hand after surgery for thumb-in-palm deformity in children with spastic hemiplegic CP. Moreover, children with unilateral CP due to congenital lesions have been shown to have unimanual and bimanual performance strongly determined by distal strength [17]. Gaillard et al*.* have also suggested that small deviations in the proximal segments may result in greater deviations in the more distal segments, which is a plausible explanation [53]. In agreement with Mailleux et al., motion analysis in children with unilateral CP and grasping ability should be considered complementary to the clinical scale to capture proximal motor deficits [16].

On a side note, the AHA is not correlated to all the distal movement since only moderate [16] or low [54] correlations were found for all forearm pronation kinematics. This is interesting because the AHA item "Moves forearm" is actually a misfit item of the AHA 5.0 internal consistency that was retained for its clinical relevance [134]. Therefore, it is not surprising that pronation kinematics does not correlate well with bimanual performance.

Regarding patients with bilateral impairments, there is limited research available. However, it can be expected that proximal impairments may play a more important role in the deficiency, since children with a candelabra pattern, as described in the Bard-Chaleat classification [135], are limited by having both their hands outside the bimanual area. Future studies will have to investigate the relationship between 3D motion analysis parameters and clinical scales for this population.

A limitation of the present review is that the correlation of parameters with clinical outcomes is strongly influenced by the task being measured, but this aspect was not investigated. The variety of task types studied in the literature, as well as the variety of existing tasks, makes any further analysis on this basis complex.

To guide therapeutic decisions effectively, a framework that links clinical questions to specific parameters and tasks for measurement and the results to appropriate intervention is required. However, few studies defined clear clinical questions, making it challenging to determine appropriate protocols, prioritize parameters, and effectively link the data obtained to therapeutic projects. The protocols included in the analysis mainly aimed to understand pathological movement in comparison to healthy movement, retrospectively investigate intervention effects, or statistically validate study design repeatability. As a result, while these protocols accurately distinguish between pathological and healthy movement, they do not provide direct therapeutic guidance. Thus, given this limited use of kinematics in decision making, it is not surprising that healthcare facilities may be reluctant to invest time and funding in expensive motion capture equipment. This is particularly true when the population being studied has been thoroughly assessed using clinical assessment tools.

## Conclusion

Although a large number of protocols are available in the literature, further studies are needed before the use of quantified motion analysis can be considered in clinical practice. Existing protocols primarily focus on populations that have been extensively studied through clinical assessments. These protocols typically rely on costly and resource-intensive state-of-the-art equipment. Furthermore, they often involve unimanual tasks that are overly standardized, thereby limiting their ability to provide clinicians with insights into participants' everyday arm use. Thus, the time and resources required to develop and use such protocols outweigh their benefits. It would be interesting to explore the links between outcome parameters and therapeutic guidance in order to integrate quantified movement assessment into a treatment pathway. This review also raises the question of the feasibility of performance measurement instrumentation; whether it is possible to find a compromise between the methodological requirements of quantified movement analysis and the flexibility of performance evaluation.

### Supplementary Information

**Additional file 1: Table S1.** Results of the quality assessment of included studies.

**Additional file 2: Table S2.** Data-table resulting from the data extraction.

**Additional file 3: Table S3.** Moderate to excellent correlations between kinematic parameters and clinical outcomes for given tasks.

## Data Availability

All data generated or analysed during this study are included in this published article and its Additional files.

## Abbreviations

AHA: Assisting Hand Assessment
BoHA: Both Hand Assessment
CP: Cerebral Palsy
GMFCS: Gross Motor Fonction Classification System
ICF: International Classification System
MACS: Manual Ability Classification System
PICOS: Participant, Intervention, Comparaison, Outcom, Study design
