# Supplementary material for: A systematic review of instrumented assessments for upper limb function in cerebral palsy: current limitations and future directions
Source: J Neuroeng Rehabil. 2024 Apr 16;21:56. doi: 10.1186/s12984-024-01353-6 (PMC11020208; doi:10.1186/s12984-024-01353-6)
Supplement: Supplementary file 1 — Additional file 1: Table S1. Results of the quality assessment of included studies. [file 12984_2024_1353_MOESM1_ESM.docx]

**Table S1 – Results of the quality assessment of included studies.** Each question was initially rated zero (missing information) or one (information provided). The finally score was calculated as the mean score per category times their weight and displayed out of 20. Studies that received a final score lower than 10 were excluded from the review (articles with a score ≤10 have been highlighted). The total score (%) for each criterion across all articles is also provided in the last row of the table.

|  | **Methodology** | | | | | | | | **Study Design** | | | | | **Population** | | | | **Reliability** | | | | | **Disc. & Eco** | | |  | |
| --- | --- | --- | --- | --- | --- | --- | --- | --- | --- | --- | --- | --- | --- | --- | --- | --- | --- | --- | --- | --- | --- | --- | --- | --- | --- | --- | --- |
| **Weight** | **3** | | | | | | | | **3** | | | | | **3** | | | | **2** | | | | | **1** | | |  |  |
| **Studies** | **C1** | **C2** | **C3** | **C4** | **C5** | **C6** | **C7** | **C8** | | **C9** | **C10** | **C11** | **C12** | | **C13** | **C14** | **C15** | | **C16** | **C17** | **C18** | **C19** | | **C20** | **Score** | |  |
| Aboelnasr et al., 2017 | 1 | 1 | 0 | 1 | 0 | 1 | 1 | 1 | | 1 | 1 | 1 | 1 | | 0 | 0 | 0 | | 0 | 0 | 0 | 0 | | 0 | 10 | |  |
| Artilheiro et al., 2014 | 1 | 1 | 0 | 1 | 0 | 1 | 1 | 1 | | 1 | 1 | 1 | 1 | | 1 | 1 | 1 | | 0 | 0 | 0 | 0 | | 1 | 14 | |  |
| Brochard et al., 2012 | 1 | 1 | 0 | 1 | 0 | 1 | 1 | 0 | | 1 | 1 | 1 | 1 | | 0 | 1 | 0 | | 0 | 0 | 0 | 0 | | 0 | 10 | |  |
| Brunstrom et al., 2000 | 0 | 0 | 0 | 0 | 0 | 0 | 0 | 0 | | 0 | 1 | 1 | 1 | | 1 | 0 | 0 | | 0 | 0 | 1 | 0 | | 0 | 5 | |  |
| Burtner et al., 2014 | 1 | 1 | 1 | 1 | 0 | 1 | 1 | 0 | | 0 | 1 | 1 | 1 | | 1 | 1 | 0 | | 0 | 0 | 0 | 0 | | 0 | 11 | |  |
| Butler et al., 2012 | 1 | 1 | 0 | 1 | 1 | 0 | 1 | 1 | | 1 | 1 | 1 | 1 | | 1 | 1 | 1 | | 0 | 0 | 0 | 1 | | 1 | 15 | |  |
| Butler et al., 2010 | 1 | 1 | 0 | 1 | 0 | 1 | 1 | 1 | | 1 | 1 | 1 | 1 | | 1 | 0 | 0 | | 0 | 0 | 0 | 0 | | 1 | 12 | |  |
| Cabral‑Sequeira et al., 2016 | 1 | 1 | 0 | 1 | 0 | 0 | 1 | 0 | | 1 | 1 | 1 | 1 | | 0 | 0 | 0 | | 0 | 0 | 1 | 0 | | 0 | 9 | |  |
| Cacioppo et al., 2022 | 1 | 1 | 0 | 1 | 1 | 1 | 1 | 1 | | 1 | 1 | 1 | 1 | | 0 | 0 | 0 | | 0 | 0 | 0 | 1 | | 1 | 12 | |  |
| Cacioppo et al., 2020 | 1 | 1 | 0 | 1 | 1 | 1 | 1 | 1 | | 1 | 1 | 1 | 1 | | 0 | 1 | 1 | | 1 | 0 | 0 | 1 | | 1 | 16 | |  |
| Camerota et al., 2014 | 1 | 1 | 0 | 0 | 0 | 0 | 1 | 1 | | 1 | 0 | 1 | 1 | | 0 | 0 | 0 | | 0 | 0 | 1 | 0 | | 0 | 8 | |  |
| Chang et al., 2005 | 1 | 1 | 0 | 1 | 0 | 1 | 1 | 1 | | 1 | 1 | 1 | 1 | | 0 | 0 | 0 | | 0 | 0 | 0 | 0 | | 0 | 10 | |  |
| Chen et al., 2013 | 1 | 1 | 0 | 1 | 1 | 1 | 0 | 1 | | 1 | 1 | 1 | 1 | | 1 | 1 | 0 | | 0 | 0 | 1 | 0 | | 0 | 13 | |  |
| Chen et al., 2014 | 1 | 1 | 0 | 1 | 1 | 1 | 1 | 1 | | 1 | 0 | 1 | 1 | | 1 | 0 | 0 | | 0 | 0 | 1 | 0 | | 0 | 12 | |  |
| Chen et al., 2016 | 1 | 1 | 0 | 1 | 1 | 1 | 1 | 0 | | 1 | 0 | 1 | 1 | | 1 | 0 | 0 | | 0 | 0 | 1 | 0 | | 0 | 11 | |  |
| Chen et al., 2015 | 1 | 1 | 0 | 0 | 1 | 1 | 1 | 0 | | 1 | 1 | 1 | 1 | | 0 | 1 | 0 | | 0 | 0 | 1 | 0 | | 1 | 12 | |  |
| Chen et al., 2007 | 1 | 1 | 0 | 0 | 1 | 1 | 0 | 1 | | 1 | 1 | 1 | 1 | | 1 | 0 | 1 | | 0 | 0 | 1 | 0 | | 1 | 13 | |  |
| Choi et al., 2021 | 1 | 1 | 0 | 1 | 1 | 1 | 1 | 0 | | 0 | 0 | 0 | 1 | | 0 | 1 | 0 | | 0 | 0 | 1 | 0 | | 1 | 8 | |  |
| Cimolin et al., 2019 | 1 | 1 | 0 | 1 | 1 | 1 | 1 | 1 | | 1 | 0 | 1 | 1 | | 0 | 1 | 0 | | 0 | 0 | 1 | 0 | | 0 | 12 | |  |
| Coluccini et al., 2007 | 1 | 1 | 0 | 1 | 0 | 0 | 1 | 1 | | 1 | 1 | 1 | 1 | | 1 | 0 | 0 | | 0 | 0 | 0 | 0 | | 0 | 10 | |  |
| de Bruin et al., 2013 | 1 | 1 | 0 | 1 | 0 | 1 | 1 | 1 | | 1 | 1 | 0 | 1 | | 0 | 1 | 0 | | 0 | 0 | 0 | 0 | | 0 | 10 | |  |
| Domellof et al., 2009 | 1 | 1 | 0 | 1 | 0 | 0 | 1 | 1 | | 1 | 1 | 1 | 1 | | 0 | 1 | 0 | | 0 | 0 | 0 | 1 | | 0 | 11 | |  |
| Eliott et al., 2011a | 1 | 1 | 0 | 1 | 0 | 0 | 1 | 0 | | 0 | 0 | 0 | 1 | | 1 | 1 | 0 | | 0 | 0 | 1 | 0 | | 0 | 8 | |  |
| Eliott et al., 2011b | 1 | 1 | 0 | 1 | 0 | 0 | 1 | 0 | | 0 | 1 | 1 | 1 | | 1 | 1 | 0 | | 0 | 0 | 1 | 0 | | 0 | 10 | |  |
| Fitoussi et al., 2011 | 1 | 1 | 0 | 1 | 1 | 1 | 1 | 1 | | 0 | 0 | 1 | 1 | | 1 | 0 | 0 | | 0 | 0 | 1 | 0 | | 1 | 11 | |  |
| Fitoussi et al., 2006 | 1 | 1 | 0 | 1 | 0 | 1 | 1 | 1 | | 0 | 0 | 1 | 1 | | 0 | 1 | 1 | | 0 | 0 | 0 | 0 | | 0 | 10 | |  |
| Francisco-Martinez et al., 2022 | 1 | 1 | 0 | 1 | 0 | 0 | 1 | 0 | | 1 | 1 | 1 | 1 | | 1 | 1 | 1 | | 0 | 0 | 0 | 0 | | 0 | 11 | |  |
| Furaya et al., 2015 | 1 | 1 | 0 | 1 | 1 | 0 | 1 | 1 | | 1 | 1 | 1 | 1 | | 1 | 1 | 0 | | 0 | 0 | 0 | 1 | | 0 | 13 | |  |
| Gaillard et al., 2020 | 1 | 1 | 0 | 1 | 1 | 1 | 1 | 1 | | 1 | 1 | 1 | 1 | | 0 | 1 | 0 | | 0 | 0 | 0 | 1 | | 1 | 14 | |  |
| Gaillard et al., 2018 | 1 | 1 | 1 | 1 | 1 | 1 | 1 | 1 | | 1 | 1 | 1 | 1 | | 0 | 1 | 0 | | 0 | 0 | 0 | 1 | | 0 | 14 | |  |
| Hervey et al., 2013 | 1 | 1 | 0 | 0 | 0 | 0 | 0 | 0 | | 1 | 1 | 1 | 1 | | 0 | 1 | 0 | | 0 | 0 | 0 | 0 | | 0 | 7 | |  |
| Huang et al., 2014 | 1 | 1 | 0 | 1 | 0 | 0 | 1 | 0 | | 0 | 1 | 1 | 1 | | 1 | 0 | 0 | | 0 | 0 | 0 | 0 | | 1 | 8 | |  |
| Hung et al., 2020 | 1 | 1 | 0 | 1 | 0 | 1 | 1 | 1 | | 1 | 1 | 1 | 1 | | 1 | 1 | 0 | | 0 | 0 | 1 | 0 | | 1 | 14 | |  |
| Hung et al., 2019 | 1 | 1 | 0 | 1 | 1 | 1 | 1 | 0 | | 1 | 1 | 1 | 1 | | 1 | 1 | 0 | | 0 | 0 | 0 | 1 | | 1 | 13 | |  |
| Hung et al., 2018 | 1 | 1 | 0 | 0 | 0 | 1 | 1 | 1 | | 1 | 1 | 0 | 1 | | 1 | 1 | 0 | | 0 | 0 | 0 | 0 | | 1 | 10 | |  |
| Hung et al., 2017 | 1 | 1 | 0 | 1 | 0 | 1 | 1 | 1 | | 1 | 1 | 1 | 1 | | 1 | 1 | 0 | | 0 | 0 | 1 | 0 | | 1 | 13 | |  |
| Hurvitz et al., 2003 | 1 | 1 | 0 | 1 | 0 | 1 | 1 | 0 | | 0 | 1 | 0 | 1 | | 1 | 0 | 0 | | 0 | 0 | 1 | 0 | | 0 | 9 | |  |
| Jaspers et al., 2011c | 1 | 1 | 0 | 1 | 0 | 1 | 1 | 1 | | 1 | 0 | 1 | 1 | | 0 | 1 | 0 | | 0 | 0 | 0 | 0 | | 0 | 10 | |  |
| Jaspers et al., 2011a | 1 | 1 | 0 | 1 | 0 | 0 | 1 | 1 | | 1 | 1 | 1 | 1 | | 0 | 1 | 1 | | 1 | 0 | 0 | 0 | | 0 | 12 | |  |
| Jaspers et al., 2011b | 1 | 1 | 0 | 1 | 1 | 0 | 0 | 1 | | 1 | 0 | 1 | 1 | | 0 | 1 | 1 | | 0 | 0 | 0 | 1 | | 0 | 11 | |  |
| Johansson et al., 2012 | 1 | 1 | 0 | 1 | 0 | 0 | 1 | 0 | | 1 | 1 | 1 | 1 | | 1 | 1 | 0 | | 0 | 0 | 1 | 0 | | 0 | 11 | |  |
| Johansson et al., 2014 | 1 | 1 | 0 | 1 | 0 | 1 | 1 | 0 | | 1 | 1 | 1 | 1 | | 0 | 1 | 0 | | 0 | 0 | 1 | 0 | | 0 | 11 | |  |
| Ju et al., 2010 | 1 | 1 | 0 | 1 | 1 | 1 | 1 | 1 | | 1 | 1 | 1 | 1 | | 1 | 1 | 0 | | 0 | 0 | 0 | 1 | | 0 | 14 | |  |
| Keller et al., 2017 | 1 | 1 | 0 | 1 | 1 | 1 | 1 | 0 | | 1 | 1 | 1 | 1 | | 1 | 1 | 0 | | 0 | 0 | 1 | 0 | | 1 | 13 | |  |
| Kim et al., 2012 | 1 | 1 | 0 | 1 | 0 | 1 | 1 | 1 | | 0 | 0 | 1 | 1 | | 0 | 1 | 0 | | 0 | 0 | 1 | 0 | | 0 | 10 | |  |
| Klotz et al., 2014 | 1 | 1 | 0 | 1 | 1 | 1 | 1 | 0 | | 0 | 1 | 0 | 1 | | 0 | 1 | 0 | | 0 | 0 | 0 | 1 | | 1 | 10 | |  |
| Kluzik et al., 1990 | 1 | 1 | 0 | 1 | 0 | 0 | 0 | 0 | | 1 | 1 | 1 | 1 | | 1 | 0 | 0 | | 0 | 0 | 1 | 0 | | 0 | 8 | |  |
| Krasovsky et al., 2021 | 1 | 1 | 0 | 1 | 1 | 1 | 1 | 0 | | 1 | 1 | 1 | 0 | | 0 | 1 | 1 | | 0 | 0 | 0 | 0 | | 1 | 11 | |  |
| Krebs et al., 2012 | 1 | 1 | 0 | 1 | 1 | 0 | 0 | 0 | | 1 | 1 | 0 | 1 | | 0 | 0 | 0 | | 0 | 0 | 1 | 0 | | 1 | 9 | |  |
| Kukke et al., 2016 | 1 | 1 | 0 | 1 | 1 | 1 | 1 | 1 | | 1 | 1 | 1 | 1 | | 1 | 1 | 0 | | 0 | 0 | 0 | 1 | | 0 | 14 | |  |
| Langan et al., 2010 | 1 | 1 | 0 | 1 | 0 | 1 | 1 | 1 | | 1 | 1 | 1 | 1 | | 0 | 1 | 0 | | 0 | 0 | 0 | 0 | | 0 | 11 | |  |
| Larson et al., 2006 | 1 | 1 | 0 | 0 | 0 | 1 | 1 | 0 | | 1 | 1 | 1 | 1 | | 1 | 0 | 0 | | 0 | 0 | 0 | 0 | | 0 | 8 | |  |
| Lee et al., 2013 | 1 | 1 | 0 | 1 | 1 | 1 | 1 | 1 | | 1 | 1 | 1 | 1 | | 0 | 1 | 0 | | 0 | 0 | 1 | 0 | | 0 | 13 | |  |
| Lempereur et al., 2012 | 1 | 1 | 0 | 1 | 0 | 0 | 1 | 0 | | 1 | 0 | 1 | 1 | | 0 | 1 | 1 | | 0 | 0 | 0 | 0 | | 0 | 9 | |  |
| Lunardini et al., 2015 | 1 | 1 | 0 | 1 | 1 | 1 | 1 | 1 | | 1 | 1 | 1 | 1 | | 1 | 1 | 0 | | 0 | 0 | 0 | 1 | | 0 | 14 | |  |
| Machado et al., 2019 | 1 | 1 | 0 | 1 | 0 | 0 | 1 | 1 | | 1 | 1 | 1 | 1 | | 1 | 1 | 0 | | 0 | 0 | 0 | 0 | | 1 | 11 | |  |
| Mackey et al., 2008 | 1 | 1 | 0 | 1 | 1 | 1 | 1 | 0 | | 1 | 1 | 1 | 1 | | 0 | 0 | 0 | | 0 | 0 | 1 | 0 | | 0 | 11 | |  |
| Mackey et al., 2006 | 1 | 1 | 0 | 1 | 0 | 1 | 1 | 0 | | 1 | 1 | 0 | 1 | | 0 | 0 | 0 | | 0 | 0 | 0 | 0 | | 0 | 8 | |  |
| Mackey et al., 2005 | 1 | 1 | 0 | 1 | 0 | 1 | 1 | 0 | | 1 | 1 | 1 | 1 | | 0 | 0 | 1 | | 1 | 0 | 0 | 0 | | 0 | 11 | |  |
| Mailleux et al., 2017 | 1 | 1 | 0 | 1 | 1 | 1 | 1 | 1 | | 1 | 1 | 1 | 1 | | 1 | 1 | 0 | | 0 | 0 | 0 | 1 | | 0 | 14 | |  |
| Masia et al., 2011 | 1 | 1 | 0 | 0 | 0 | 0 | 1 | 0 | | 1 | 1 | 1 | 1 | | 0 | 1 | 0 | | 0 | 0 | 0 | 0 | | 0 | 8 | |  |
| Moura et al., 2017 | 1 | 1 | 0 | 1 | 0 | 1 | 1 | 1 | | 1 | 1 | 1 | 1 | | 1 | 1 | 0 | | 0 | 0 | 1 | 0 | | 0 | 13 | |  |
| Mutalib et al., 2019 | 1 | 1 | 0 | 1 | 0 | 0 | 1 | 1 | | 0 | 1 | 1 | 1 | | 1 | 1 | 0 | | 0 | 0 | 0 | 0 | | 1 | 10 | |  |
| Mutassarts et al., 2004 | 1 | 1 | 0 | 1 | 1 | 0 | 1 | 1 | | 1 | 1 | 0 | 1 | | 1 | 0 | 0 | | 0 | 0 | 0 | 0 | | 0 | 10 | |  |
| Nicholson et al., 2001 | 1 | 1 | 0 | 1 | 1 | 0 | 1 | 1 | | 1 | 1 | 0 | 1 | | 1 | 0 | 0 | | 0 | 0 | 1 | 0 | | 0 | 11 | |  |
| Pons et al., 2017 | 1 | 1 | 0 | 1 | 1 | 1 | 1 | 1 | | 0 | 1 | 1 | 1 | | 1 | 1 | 0 | | 0 | 0 | 0 | 0 | | 1 | 12 | |  |
| Preston et al., 2014 | 1 | 1 | 0 | 0 | 0 | 0 | 0 | 0 | | 1 | 1 | 1 | 1 | | 0 | 1 | 0 | | 0 | 0 | 0 | 0 | | 0 | 7 | |  |
| Qiu et al., 2009 | 1 | 1 | 0 | 0 | 1 | 1 | 0 | 0 | | 1 | 1 | 0 | 1 | | 1 | 1 | 0 | | 0 | 0 | 1 | 0 | | 1 | 11 | |  |
| Rameckers et al., 2010 | 1 | 1 | 0 | 1 | 1 | 0 | 1 | 0 | | 1 | 1 | 1 | 1 | | 1 | 1 | 0 | | 0 | 0 | 1 | 1 | | 0 | 13 | |  |
| Rameckers et al., 2007 | 1 | 1 | 0 | 1 | 0 | 1 | 1 | 0 | | 1 | 1 | 1 | 1 | | 1 | 1 | 0 | | 1 | 0 | 1 | 0 | | 0 | 13 | |  |
| Raouafi et al., 2018 | 1 | 1 | 0 | 1 | 0 | 0 | 1 | 0 | | 1 | 1 | 1 | 1 | | 1 | 1 | 0 | | 0 | 0 | 0 | 1 | | 0 | 11 | |  |
| Reid et al., 1992b | 1 | 1 | 0 | 1 | 0 | 0 | 1 | 1 | | 1 | 1 | 1 | 1 | | 1 | 0 | 0 | | 0 | 0 | 1 | 0 | | 0 | 11 | |  |
| Reid et al., 1992a | 1 | 1 | 0 | 0 | 0 | 0 | 1 | 1 | | 1 | 1 | 1 | 1 | | 1 | 0 | 0 | | 0 | 0 | 1 | 0 | | 0 | 10 | |  |
| Reid et al., 2010 | 1 | 1 | 0 | 1 | 0 | 0 | 1 | 0 | | 1 | 0 | 1 | 1 | | 0 | 1 | 1 | | 1 | 0 | 0 | 0 | | 0 | 10 | |  |
| Ricken et al., 2005 | 1 | 1 | 0 | 1 | 0 | 0 | 1 | 1 | | 1 | 1 | 1 | 1 | | 1 | 0 | 0 | | 0 | 0 | 0 | 0 | | 1 | 10 | |  |
| Rigoldi et al., 2012 | 1 | 1 | 0 | 1 | 0 | 0 | 1 | 1 | | 1 | 1 | 1 | 1 | | 1 | 0 | 0 | | 0 | 0 | 0 | 0 | | 0 | 10 | |  |
| Ronnqvist et al., 2007 | 1 | 1 | 0 | 1 | 0 | 1 | 1 | 1 | | 1 | 1 | 1 | 1 | | 0 | 1 | 0 | | 0 | 0 | 0 | 1 | | 0 | 12 | |  |
| Rudisch et al., 2016 | 1 | 1 | 0 | 1 | 1 | 1 | 1 | 1 | | 1 | 1 | 1 | 1 | | 0 | 1 | 0 | | 0 | 0 | 0 | 1 | | 1 | 14 | |  |
| Sandlund et al., 2014 | 1 | 1 | 0 | 1 | 0 | 0 | 1 | 0 | | 1 | 1 | 1 | 1 | | 1 | 1 | 0 | | 0 | 0 | 1 | 0 | | 1 | 12 | |  |
| Sarcher et al., 2015 | 1 | 1 | 0 | 1 | 0 | 1 | 1 | 1 | | 1 | 1 | 1 | 1 | | 1 | 1 | 1 | | 0 | 0 | 0 | 0 | | 0 | 13 | |  |
| Schneiberg et al., 2010 | 1 | 1 | 1 | 1 | 0 | 1 | 1 | 1 | | 1 | 1 | 1 | 1 | | 1 | 1 | 1 | | 0 | 0 | 0 | 0 | | 0 | 14 | |  |
| Shim et al., 2022 | 1 | 1 | 0 | 1 | 1 | 1 | 0 | 1 | | 0 | 1 | 1 | 1 | | 0 | 1 | 0 | | 0 | 0 | 0 | 1 | | 1 | 12 | |  |
| Simon-Martinez et al., 2017 | 1 | 1 | 0 | 1 | 1 | 1 | 1 | 1 | | 1 | 1 | 1 | 1 | | 0 | 1 | 0 | | 0 | 0 | 0 | 0 | | 0 | 12 | |  |
| Simon-Martinez et al., 2020 | 1 | 1 | 1 | 1 | 1 | 1 | 1 | 1 | | 1 | 1 | 1 | 1 | | 0 | 1 | 0 | | 0 | 0 | 0 | 0 | | 0 | 13 | |  |
| Soares et al., 2019 | 1 | 1 | 0 | 1 | 1 | 1 | 1 | 1 | | 1 | 1 | 1 | 1 | | 0 | 0 | 0 | | 0 | 0 | 0 | 0 | | 0 | 11 | |  |
| Sohn et al., 2019 | 1 | 1 | 0 | 0 | 0 | 0 | 1 | 0 | | 1 | 1 | 1 | 0 | | 1 | 0 | 0 | | 0 | 0 | 0 | 0 | | 1 | 8 | |  |
| Steenbergen et al., 2007 | 1 | 1 | 0 | 1 | 0 | 1 | 1 | 0 | | 1 | 1 | 1 | 1 | | 1 | 0 | 0 | | 0 | 0 | 0 | 0 | | 0 | 10 | |  |
| Sugden et al., 1995 | 1 | 1 | 0 | 0 | 0 | 0 | 1 | 1 | | 1 | 1 | 1 | 1 | | 0 | 0 | 0 | | 0 | 0 | 0 | 0 | | 0 | 8 | |  |
| Surkar et al., 2019 | 1 | 1 | 1 | 1 | 1 | 1 | 1 | 1 | | 1 | 1 | 1 | 1 | | 0 | 1 | 0 | | 0 | 0 | 0 | 0 | | 0 | 13 | |  |
| Trac et al., 2018 | 1 | 1 | 0 | 1 | 0 | 0 | 1 | 0 | | 0 | 1 | 1 | 1 | | 0 | 0 | 0 | | 0 | 0 | 0 | 0 | | 1 | 8 | |  |
| Utlley et al., 2004 | 1 | 1 | 0 | 1 | 0 | 0 | 1 | 1 | | 0 | 1 | 0 | 1 | | 1 | 0 | 0 | | 0 | 1 | 0 | 0 | | 0 | 9 | |  |
| Van der Heide et al., 2005 | 1 | 1 | 0 | 1 | 1 | 0 | 1 | 0 | | 0 | 1 | 1 | 1 | | 1 | 0 | 0 | | 0 | 0 | 0 | 1 | | 0 | 10 | |  |
| Van Thiel et al., 2002 | 1 | 1 | 0 | 1 | 0 | 0 | 1 | 0 | | 1 | 1 | 1 | 1 | | 0 | 0 | 0 | | 0 | 0 | 0 | 0 | | 0 | 8 | |  |
| Vanmechelen et al., 2022 | 1 | 1 | 0 | 1 | 0 | 1 | 1 | 1 | | 1 | 1 | 1 | 1 | | 1 | 1 | 1 | | 0 | 0 | 0 | 0 | | 0 | 13 | |  |
| Volman et al., 2002 | 1 | 1 | 0 | 1 | 0 | 0 | 1 | 1 | | 1 | 1 | 1 | 1 | | 1 | 0 | 0 | | 0 | 0 | 0 | 0 | | 0 | 10 | |  |
| Weightman et al., 2011 | 1 | 1 | 0 | 1 | 0 | 1 | 1 | 0 | | 0 | 1 | 0 | 0 | | 0 | 1 | 0 | | 0 | 0 | 1 | 0 | | 0 | 8 | |  |
| Weigthmann et al., 2014 | 1 | 1 | 0 | 1 | 0 | 0 | 1 | 0 | | 0 | 1 | 1 | 1 | | 1 | 1 | 0 | | 0 | 0 | 0 | 0 | | 1 | 10 | |  |
| *% per criteria* | *98%* | *98%* | *5%* | *86%* | *39%* | *58%* | *88%* | *59%* | | *79%* | *84%* | *86%* | *96%* | | *54%* | *63%* | *15%* | | *5%* | *1%* | *34%* | *20%* | | *30%* |  | |  |

Disc. & Eco: Discriminatory Power & Ecological Validity, C1: *Objectives*, C2: *Parameters*, C3: *Sample* *Size*, C4: *Statistics*, C5: *Clinical* *Assessment*, C6: *Limits*, C7: *Literature*, C8: *Installation*, C9: *Tasks*, C10: *Equipment* C11: *Outcome* *Parameters*, C11: *Laterality*, C13: *Dominant* *Type*, C14: *Functional* *Impairment*, C15: *Test-retest Reliability*, C16: *Inter-session Reliability*, C17: *Inter-rater Reliability*, C18: *Sensibility to Change*, C19: *Discriminatory Power*, C20: *Semi-ecological*
